# Non-lytic protein secretion by virulence-associated Type 10 Secretion Systems in *Salmonella enterica*

**DOI:** 10.3389/fmicb.2026.1809111

**Published:** 2026-06-02

**Authors:** Srujita Mahankali, Tobias Geiger

**Affiliations:** Chair of Medical Microbiology and Hospital Hygiene, Max von Pettenkofer-Institute, Ludwig-Maximilians-Universität München (LMU Munich), Munich, Germany

**Keywords:** chitinase A, *Salmonella Typhi* (*S. Typhi*), secretion systems, Type 10 Secretion System, typhoid toxin, typhoidal and non-typhoidal *Salmonella* serovars, virulence factors

## Abstract

**Introduction:**

Type 10 Secretion Systems (T10SSs) are critical virulence determinants in many bacterial pathogens and are evolutionarily derived from phage-associated lytic cassettes. In *Salmonella enterica*, two distinct T10SSs mediate the export of key virulence factors: typhoid toxin and cytolysin A in *S. enterica* serovar Typhi, the causative agent of typhoid fever, and the invasion-associated chitinase A in non-typhoidal *S. enterica* serovar Typhimurium, a major cause of severe salmonellosis worldwide. These systems respond to distinct environmental cues, with the typhoidal T10SS induced intracellularly within the *Salmonella*-containing vacuole and the non-typhoidal T10SS activated extracellularly upon contact with intestinal epithelial cells.

**Results and methods:**

Here, we show that both T10SSs are tightly regulated by specific transcriptional regulators and are expressed exclusively under physiologically relevant inducing conditions. Under these conditions, T10SS activation results in robust expression of the secretion machinery at both the promoter and the protein levels. Population- and single-cell analyses further reveal that induction by environmental cues leads to activation of T10SS expression in the majority of the bacterial population in both *S. Typhi* and *S. Typhimurium*. Importantly, single-cell fluorescence and viability analyses demonstrate that secretion occurs without detectable bacterial lysis, indicating that T10SSs function as non-lytic secretion systems.

**Discussion:**

Despite their origin from phage-derived lytic modules, our data show that *Salmonella* T10SSs have been repurposed into actively secreting systems that export virulence factors from viable bacterial cells under tight regulatory control. Together, these findings provide new insight into the regulation and function of T10SSs in *Salmonella enterica* and highlight an evolutionary adaptation of phage-derived machinery into specialized secretion systems essential for bacterial virulence.

## Introduction

*Salmonella enterica* is a prevalent Gram-negative pathogen, capable of infecting a broad range of hosts. *Salmonella* serovars are broadly categorized into typhoidal and non-typhoidal strains, varying in their host specificity and severity of disease. Typhoidal *S.* Typhi and *S.* Paratyphi A are human-restricted pathogens responsible for systemic, enteric fever, unlike non-typhoidal serovars that affect a broad range of hosts and lead to acute enteritis (Wang et al., 2023; Rosselin et al., 2012; LaRock et al., 2015). These differences in behavior of typhoidal and non-typhoidal *Salmonella* have been attributed to the differences in virulence factors expressed, such as the higher expression of *fepE* observed in *S.* Paratyphi A in comparison to *S.* Typhimurium leading to the generation of very long O-antigen chains that help evasion of immune response (Mylona et al., 2021). Another such virulence factor is the typhoid toxin which is specific to typhoidal serovars *S.* Paratyphi A and *S.* Typhi. The typhoid toxin is an A_2_B_5_ type toxin that is composed of CdtB, PltA, and PltB subunits, which play a role in the intoxication of host cells, DNA damage and cell cycle arrest (Spanò et al., 2008). Secretion of this toxin in *S.* Typhi has previously been shown to occur via a Type 10 Secretion System (T10SS) (Geiger et al., 2018, 2020).

The T10SS is a phage derived, two-step secretion system described in *Serratia* for the secretion of chitinase in the absence of the Type 2 Secretion System. Initial studies performed in *S. marcescens* DB10 found the *chiWXYZ* operon encoding the holin ChiW, peptidoglycan hydrolase ChiX and spanins ChiY and ChiZ, to be responsible for this secretion. The *chiWXYZ* operon was also found to be similar in sequence and related to the lambda phage lysis cassette (Hamilton et al., 2014; Palmer et al., 2021).

Two such T10SSs have been described in *Salmonella enterica*, extracellularly in non-typhoidal *Salmonella* for the secretion of chitinase during invasion of intestinal epithelial cells (IECs), and intracellularly by typhoidal serovars for the secretion of cytolysin A or typhoid toxin within the *Salmonella* containing vacuole (SCV) (Krone et al., 2023, 2024; Geiger et al., 2020). The chitinase operon in *Salmonella* consists of a holin chiH, the peptidoglycan hydrolase chiP, chitinase A chiA and is tightly regulated by the Tox-R-like regulator chiR. Upon contact with mucin-expressing polarized intestinal epithelial cells (IECs), the ChiR regulator is activated, leading to induction of the T10SS. Under these conditions, ChiH facilitates the translocation of ChiP to the periplasm, where it cleaves the peptidoglycan layer, thereby enabling the secretion of chitinase A (Krone et al., 2023). Notably, ChiP lacks a signal sequence for secretion and therefore depends on the holin to traverse the inner membrane and access the periplasm.

Within the SCV, the T10SS has also been described in the secretion of the typhoid toxin, and cytolysin A. Typhoid toxin secretion is executed by a holin, the peptidoglycan hydrolase TtsA and a LD-transpeptidase YcbB. Individual subunits of the toxin are transported to the periplasm by the canonical Sec machinery, where the toxin is assembled. The LD-transpeptidase YcbB is similarly translocated to the periplasm where it modifies the peptidoglycan layer at the poles, converting the common 4-3 or DD crosslinks to 3-3 or LD crosslinks (Geiger et al., 2018). The translocation of TtsA to the periplasm is facilitated by a yet unidentified holin, as TtsA, similar to ChiP, lacks a signal sequence for secretion. Once in the periplasm, TtsA selectively cleaves these DD crosslinks allowing for the toxin to cross from the cis to trans side of the peptidoglycan layer. The mechanism of release of the typhoid toxin across the outer membrane is yet unknown, however sub-inhibitory concentrations of antimicrobial peptides or bile salts have been shown to trigger this release (Geiger et al., 2018, 2020; Hodak and Galán, 2013). Interestingly, TtsA has previously been shown to belong to the family of coliphage N4 Gp61 N-acetyl-muramidases along with ChiP (Stojković and Rothman-Denes, 2007).

Due to its origin from phage lysis cassettes, multiple studies have inspected if the T10SS is a lytic system allowing for release of the cargo protein by bacterial lysis. In *Yersinia entomophaga*, an insect pathogen affecting beetles, the secretion of the toxin YenTc was described to be by a lytic T10SS. The large (2.4 MDa) toxin was shown to be secreted only by a small population of cells by a spanin-mediated fusion of the inner and outer membranes leading to lysis and release of the toxin (Sitsel et al., 2024; Feldmüller et al., 2024). Conflicting reports have been published with regards to the lytic or non-lytic nature of the secretion of *Clostridium difficile* toxins A and B. Toxin release is facilitated by the action of the holin TdcE and hydrolase TdcL, however while Kordus et al. (2025) suggest that a minority subpopulation undergoes TcdE-mediated lysis to release the toxins, previous studies suggest the secretion occurs in a specific, non-lytic fashion (DiBenedetto et al., 2025; Govind and Dupuy, 2012; Kordus et al., 2025). This is also the case in *Serratia marcescens*, where early studies on the chitinase T10SS in *S. marcescens* DB10 were shown to be non-lytic. This was contradicted by a more recent study using *S. marcescens* BS303, where an arabinose-inducible ChiR strain was used under anaerobic stress conditions and lytic secretion similar to *Y. entomophaga* was observed (Costa et al., 2019; Hamilton et al., 2014; Chowdhury et al., 2025; Owen et al., 2018; Sitsel et al., 2024).

This study examines the functional nature of the two T10SSs described in *Salmonella enterica* to determine whether they function in a lytic or non-lytic manner. Using physiologically relevant inducing conditions, we first confirmed that the secretion components are sufficiently expressed under the tested conditions and are tightly regulated at both the promoter and the protein levels. We then demonstrate, using cell-based infection assays, that under these *in vivo*-like conditions, the majority of the bacterial population expresses the secretion components, during contact with polarized IECs in the case of chitinase secretion, and within the SCV in the case of typhoid toxin secretion. Importantly, we show that under these natural induction conditions, secretion occurs without detectable bacterial lysis. Together, these findings demonstrate that *Salmonella enterica* T10SSs function as non-lytic, actively secreting systems that repurpose phage-derived lysis modules to export cargo proteins from viable cells under tight regulatory control.

## Materials and methods

### Cloning/mutagenesis

The bacterial strains and plasmids used in this study are listed in Table 1. All *Salmonella* Typhimurium and Typhi strains are derived from *Salmonella enterica* serovar Typhimurium SL1344 and serovar Typhi Ty2, respectively (Hapfelmeier et al., 2005; Deng et al., 2003). All in-frame deletions were generated using an optimized site-directed scarless mutagenesis protocol (Bender et al., 2013; Hoffmann et al., 2017). Briefly, a recombinant PCR product containing a kanamycin resistance cassette and a recognition site for the meganuclease I-SceI was integrated into the chromosome using heat-induced λ Red recombinase. In a second step, the cassette was replaced with a PCR product generating a clean deletion. Mutants retaining the cassette were eliminated by double-strand breaks induced by the AHT-inducible meganuclease I-SceI.

**TABLE 1:** Bacterial strains and plasmids used in this study.

Names	Genotypes	Constructed by
Strains		
SB300	*S*. Typhimurium SL1344 WT Str^R^	Hapfelmeier et al., 2005
TG0018	*S*. Typhimurium SL1344 *chiH:3xFLAG*	This study
TG0019	*S*. Typhimurium SL1344 *chiP:3xFLAG*	This study
TG0022	*S*. Typhimurium SL1344 Δ*chiR, chiH*:3xFLAG	This study
TG0023	*S*. Typhimurium SL1344 Δ*chiR, chiP*:3xFLAG	This study
TG0008	*S*. Typhimurium SL1344 Δ*chiH*	This study
TG0009	*S*. Typhimurium SL1344 Δ*chiP*	This study
TG0064	*S*. Typhimurium SL1344 *chiH:sfGFP*	This study
TG0065	*S*. Typhimurium SL1344 *chiP:sfGFP*	This study
TG0066	*S*. Typhimurium SL1344 Δ*chiR, chiH:sfGFP*	This study
TG0067	*S*. Typhimurium SL1344 Δ*chiR, chiP:sfGFP*	This study
TG0070	*S*. Typhi Ty2 WT	Deng et al., 2003
TG0073	*S*. Typhi Ty2 *ttsA:3xFLAG*	This study
TG0081	*S*. Typhi Ty2 *ttsA:3xFLAG, ΔphoP*	This study
TG0077	*S*. Typhi Ty2 *ttsA:sfGFP*	This study
TG0080	*S*. Typhi Ty2 *ttsA:sfGFP, ΔphoP*	This study
TG0078	*S*. Typhi Ty2 Δ*ttsA*	This study
Plasmids		
pWRG167	P*_*EM7*_*:*sfgfp* in pWRG81, Ap^r^	Bender et al., 2013
pWRG717	Scarless mutagenesis template plasmid	Hoffmann et al., 2017
pWRG730	Scarless mutagenesis plasmid	Hoffmann et al., 2017
pTG0044	Δ*P:sfGFP* pWRG167 with deleted PEM7 promoter in front of sfGFP	Krone et al., 2024
pTG0076	pWRG717 for 3xFLAG epitope tagging	(Krone et al., 2024)
pTG0110	pWRG717 for sfGFP replacement mutagenesis	This study
pTG0103	pT2 plasmid with sfGFP	This study
pTG0112	pTG0103 with *ttsA promoter* (500bp) in front of sfGFP	This study
pTG0113	pTG0103 with *chiH promoter* (684bp) in front of sfGFP	This study
pTG0114	pTG0103 *with chiP promoter* (840bp) in front of sfGFP	This study

For chromosomal insertion of C-terminal 3 × FLAG epitope tags, the same optimized site-directed scarless mutagenesis protocol was used. The original template plasmid pWRG717 was modified, resulting in plasmid pTG0076, which was used as the template for chromosomal 3 × FLAG tag insertion. The same mutagenesis procedure was applied to generate sfGFP replacement mutants in the chromosome using plasmid pTG0110, which is also derived from pWRG717 (Hoffmann et al., 2017).

Primers used for cloning are listed in Supplementary Table 2. All plasmids were constructed using the Gibson assembly cloning strategy. All generated plasmids and bacterial strains were verified by nucleotide sequencing.

### Bacterial and eukaryotic cell cultures

*Salmonella* strains were routinely cultured on standard LB agar plates or in liquid LB medium (10 g/L NaCl, 10 g/L tryptone, 5 g/L yeast extract) at 37 °C with shaking. Where appropriate, antibiotics were added to bacterial cultures at the following concentrations: 50 μg/mL kanamycin (Carl Roth, Chemicals), 100 μg/mL ampicillin, or 10 μg/mL chloramphenicol (Carl Roth, Chemicals).

HeLa cells, obtained from the Leibniz Institute DSMZ-German Collection of Microorganisms and Cell Cultures GmbH, were cultured in Dulbecco’s modified Eagle’s medium (DMEM, high glucose, with glutamine; Gibco) supplemented with 10% fetal calf serum (FCS; Gibco). CaCo-2 cells were maintained in DMEM (high glucose, with glutamine; Gibco) supplemented with 10% FCS and 1 × non-essential amino acids (MEM NEAA, 100×; Gibco). For CaCo-2 cells, 1 mM sodium pyruvate (Gibco) was additionally added. All eukaryotic cells were incubated at 37 °C in a humidified atmosphere containing 5% CO_2_.

### Western blot analyses

Western blot analyses were performed as previously described [64]. For detection of CdtB or TtsA, 4 × 10^8^ bacteria were used to infect HeLa cells at an MOI of 1:50 for 24 h. Following incubation, bacteria were harvested for Western blot analysis. For colony forming units (CFU) determination, serial dilutions were plated on agar plates, yielding bacterial counts of 8 × 10^7^. For ChiA, ChiH, and ChiP expression analyses, bacteria were incubated in contact with polarized Caco-2 cells. In these experiments, 6 × 10^7^ bacteria were used and incubated for 3 h. After incubation, bacteria were harvested for Western blot analysis. CFU determination by serial dilution plating yielded bacterial counts of 1.5 × 10^8^.

Samples (bacteria pellets) for Western blot analysis (TtsA-3 × FLAG, ChiH-3 × FLAG, or ChiP-3 × FLAG) were mixed with Laemmli SDS sample buffer, boiled for 5 min at 99 °C, and separated on a 12% SDS–polyacrylamide gel. Proteins were transferred to a 0.45 μm nitrocellulose membrane (Amersham Protran, 0.45 μm NC) for 1 h at 20 V and 0.3 A. The membrane was blocked for 30 min in TBS containing 5% non-fat milk (Carl Roth, Chemicals).

The membrane was incubated overnight at 4 °C with an anti-FLAG M2 mouse monoclonal primary antibody (Sigma) diluted 1:10,000 in TBS-T supplemented with 5% non-fat milk. After washing three times with TBS-T, the membrane was incubated for 1 h at room temperature with a goat anti-mouse HRP-conjugated secondary antibody diluted 1:10,000 in TBS-T containing 5% non-fat milk. The membrane was then washed three times with TBS-T. Chemiluminescent detection was performed using Immobilon Crescendo Western HRP substrate (Millipore), and signals were recorded using a ChemiDoc imaging system (BioRad).

### Fluorescence reporter assays

For analysis of *ttsA*, *chiH*, and *chiP* promoter activities, fluorescence reporter strains carrying chromosomal *ttsA:sfGFP*, *chiH:sfGFP*, or *chiP:sfGFP* replacements were generated in *Salmonella* Typhi or Typhimurium respectively, along with the corresponding regulator deletion mutants (*phoP* or *chiR*, respectively).

#### ttsA promoter activity during infection

For analysis of *ttsA:sfGFP* reporter strains and the corresponding *phoP* deletion mutant in *S.* Typhi, HeLa cells were seeded in 24-well plates at a density of 2.0 × 105 cells per well on the day of infection. Bacteria were grown overnight in LB medium and subcultured 1:30 in LB supplemented with 0.3 M NaCl to induce SPI-1 T3SS–mediated invasion [60]. Subcultures were incubated at 37 °C with shaking until reaching an OD_600_ of 0.9. Bacteria were then diluted in DMEM and added to host cells at a multiplicity of infection (MOI) of 30 for 60 min. Cells were washed three times with DPBS (Gibco) to remove extracellular bacteria and incubated for an additional 60 min in DMEM containing 100 μg/mL gentamicin to kill remaining extracellular bacteria. The medium was subsequently replaced with DMEM containing 10 μg/mL gentamicin, and cells were incubated for 23 h.

Host cells were lysed with 1,000 μL of 0.1% Triton X-100. After 10 min of incubation, lysates were homogenized by vigorous pipetting and collected. GFP fluorescence (excitation 488 nm) was measured in 96-well plates using 100 μL aliquots of each lysate with a Clariostar plate reader (BMG Labtech).

#### chiH and chiP promoter activity *in vitro*

To analyze *chiH:sfGFP* and *chiP:sfGFP* reporter strains and the corresponding *chiR* deletion mutants in *S.* Typhimurium, bacteria were grown overnight in LB medium, washed with PBS, and subcultured in fresh LB medium at a starting OD_600_ of 0.1. After 2 h of growth (exponential growth phase), bacteria were washed with PBS and diluted 1:10 in 1% mucin extract dissolved in DMEM (porcine stomach mucin; 10% stock solution, Sigma). Cultures were incubated for 3 h, after which bacteria were pelleted and resuspended in PBS. GFP fluorescence was measured in 96-well plates using 100 μL aliquots of each bacterial suspension with a Clariostar plate reader (BMG Labtech).

#### Controls and normalization

For both experimental conditions, *S.* Typhi or *S.* Typhimurium grown in standard liquid LB medium served as negative controls. All fluorescence intensities were normalized to an equal bacterial density of 1 × 10^8^ bacteria/mL. Bacterial counts were determined by plating serial dilutions on LB agar plates to calculate CFUs. Data represent the mean values of three independent biological replicates.

### Flow cytometry analyses

Single-cell fluorescence analyses were performed to assess bacterial subpopulations. For this purpose, chromosomal replacement mutants in which the gene of interest was replaced by sfGFP (ttsA:sfGFP, chiH:sfGFP, and chiP:sfGFP) were used. In addition, wild-type bacteria harboring reporter plasmids, in which the corresponding promoter regions upstream of the genes of interest were cloned in front of the gene encoding sfGFP (ttsA-P:sfGFP, chiH-P:sfGFP, and chiP-P:sfGFP), were analyzed.

For *ttsA* analyses, intracellular bacteria of *S.* Typhi were used, whereas for *chiH* and *chiP* analyses, *S.* Typhimurium in contact with polarized CaCo-2 cells was analyzed. Bacteria were harvested either 24 h post infection or after 3 h of incubation with CaCo-2 cells. Host cells were lyzed if necessary and removed by low-speed centrifugation (500 × *g*), and bacteria were pelleted by high-speed centrifugation (13,000 rpm for 5 min). Bacterial pellets were resuspended in 4% paraformaldehyde (PFA) and fixed for 20 min. After centrifugation (10,000 × g for 5 min), pellets were washed and resuspended in PBS.

Fluorescence intensities were measured by flow cytometry using a BD FACSCanto II. Green fluorescence was excited at 488 nm. Gates were set using a promoterless sfGFP plasmid (pTG0044) or a non-fluorescent wild-type S. Typhimurium strain as negative controls, and a constitutive GFP-expressing plasmid as a positive control (pTG 0029). Data were analyzed using FlowJo software (FlowJo LLC). Data represent the mean values of three independent biological replicates.

Cell death was assessed using TO-PRO (Thermo Fisher Scientific), a membrane-impermeable nucleic acid stain that enters cells only when membrane integrity is lost. Reporter strains as described before were analyzed at the single-cell level to quantify bacterial cell death and lysis.

After cultivation of the bacteria, 24 hpi for S. Typhi (8 × 10^7^ bacteria) or 3 h co-cultivation with Caco-2 cells for *S*. Typhimurium (1.5 × 10^8^ bacteria), bacterial cultures were stained with 2 drops of ready-to-use TO-PRO for 20 min in the dark. As a positive control for cell death, bacteria were lysed by incubation in 70% isopropanol at 75 °C for 20 min. As a negative control, wild-type bacteria from a shaking subculture (1:30) incubated for 3 h at 37 °C were used. Positive and negative control samples were stained and processed in the same manner as the experimental samples. Fluorescence intensities were measured by flow cytometry in the far-red channel (emission at 650 nm) using red-laser excitation. No spectral bleed-through into the GFP channel (488 nm excitation) was detected, as confirmed by appropriate controls. Data represent the mean values of three independent biological replicates.

### Colony forming units (CFU)

Colony forming units were determined for *S.* Typhi 24 h post infection in wild-type strains and the corresponding *ttsA* clean deletion mutant, as well as for *S.* Typhimurium after 3 h of incubation in contact with polarized CaCo-2 cells in wild-type strains and the corresponding *chiH* and *chiP* clean deletion mutants. Serial dilutions were plated on LB agar plates. Data represent the mean values of three independent biological replicates.

### Statistical analyses

Data were plotted using GraphPad Prism 10 software. All data were acquired from ≥three independent biological replicates. Significance is given as follows: Not significant (ns): *p* > 0.05, *: *p* < 0.05, **: *p* < 0.01. ***: *p* < 0.001, ****: *p* < 0.0001.

### Data availability

All data generated or analyzed during this study are included in this published article and its Supplementary materials. Source data are provided with this paper.

## Results

### *In vivo*-like induction and regulatory control of Type 10 Secretion Systems in typhoidal and non-typhoidal *S. enterica*

**FIGURE 1:**
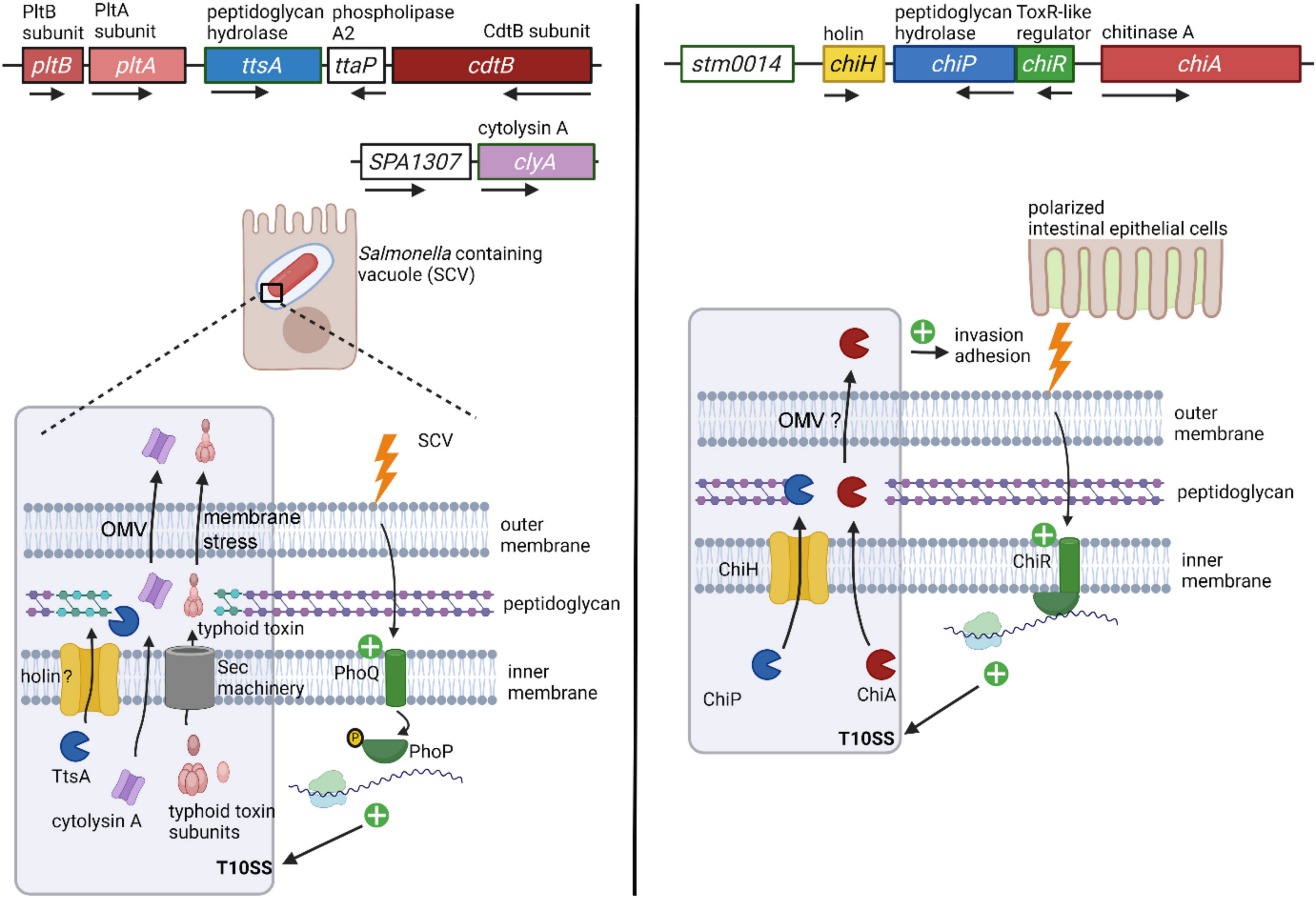
Genomic organization of distinct operons and current models of Type 10 Secretion Systems (T10SS) in *Salmonella enterica*. (Left) T10SS mediating secretion of typhoid toxin and cytolysin A in typhoidal *Salmonella enterica* serovars, such as *S*. Typhi and *S*. Paratyphi A. Induction and activation of this system occur within the *Salmonella*-containing vacuole of infected host cells via the PhoP/Q regulatory system. (Right) T10SS in non-typhoidal *S. enterica* serovar Typhimurium, induced upon contact with mucin-expressing polarized intestinal epithelial cells through the ChiR regulator. The black arrows shown beneath the genes indicate the direction of transcription. Created in BioRender. Geiger, T. (2026) https://BioRender.com/m42dwlo.

To date, two T10SSs have been described in *S. enterica*. Each system is encoded by a distinct operon that contains genes for both the secretion machinery and the corresponding secreted cargo proteins (Figure 1). Previous studies have shown that in the typhoidal serovars *S. enterica* serovar Typhi (*S*. Typhi) and Paratyphi A (*S*. Paratyphi A), T10SS expression is induced intracellularly following host cell infection. The major secretion component, TtsA, is highly expressed only after bacterial localization within the SCV and mediates the secretion of the cargo proteins typhoid toxin and cytolysin A (Geiger et al., 2018; Krone et al., 2024). In contrast, in the non-typhoidal serovar *S. enterica* serovar Typhimurium (*S*. Typhimurium), a functionally distinct T10SS operates at the intestinal mucosal interface and is responsible for the secretion of ChiA, as demonstrated by Krone et al. (2023). In both systems, tightly controlled regulatory mechanisms ensure precise induction of the secretion components and expression of the cargo proteins, preventing excessive activation. For the typhoidal T10SS, the intravacuolar activated response regulator PhoP plays a key regulatory role, whereas in the non-typhoidal T10SS the response regulator ChiR is essential for system function (Figure 1).

**FIGURE 2:**
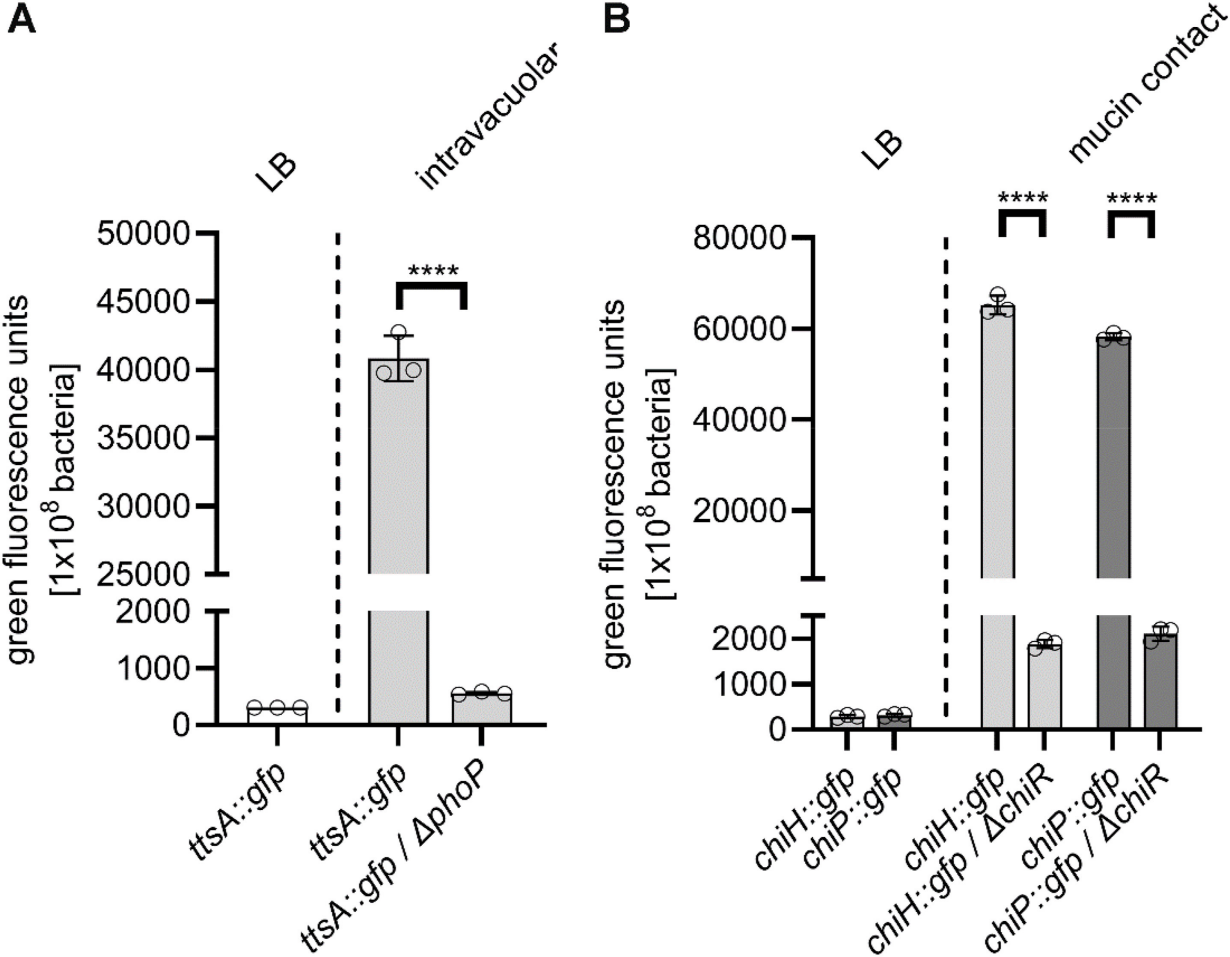
Promoter activity analyses of components of the T10SS. Reporter strains were generated by chromosomal replacement with sfGFP. **(A)** *ttsA* promoter activity was assessed using a *ttsA:sfGFP* reporter strain and a *ttsA:sfGFP*Δ*phoP* mutant. As a negative control, strains were incubated in standard LB medium. For induction, intracellular bacteria were recovered at 24 h post infection (hpi). Fluorescence measurements were normalized to a bacterial cell density of 108, as determined by CFU plating. Data represent three independent experiments and are shown as mean ± standard deviation. Statistical analysis was performed using an unpaired *t*-test; ****p < 0.001. **(B)** Promoter activities of *chiH* and *chiP* were analyzed using *chiH:sfGFP* and *chiP:sfGFP* reporter strains. LB medium served as a negative control. Induction was performed in DMEM supplemented with 1% mucin for 3 h. *chiR* mutant strains, lacking the main regulator of the T10SS in *S.* Typhimurium, were included. Fluorescence measurements were normalized to a bacterial cell density of 108, as determined by CFU plating. Data represent three independent experiments and are shown as mean ± standard deviation. Statistical analysis was performed using an unpaired *t*-test; ****p < 0.0001.

In this study, we verified that upon uptake of *S.* Typhi by host cells, the major secretion component and key effector of the secretion system, the peptidoglycan hydrolase TtsA, is induced. To assess *ttsA* expression, we generated a chromosomal reporter strain in which *ttsA* was replaced by *sfGFP*. This reporter strain was used in fluorescence-based promoter assays. While standard LB medium did not induce *ttsA* promoter activity, strong induction was observed following uptake of bacteria by host cells (Figure 2A). This induction depends on a functional PhoP regulator, as *ttsA* promoter-driven fluorescence was abolished in a Δ*phoP* mutant.

In *S.* Typhimurium, contact with polarized IECs or mucins resulted in induction of the non-typhoidal T10SS. Promoter assays using *chiH:sfGFP* and *chiP:sfGFP* reporter strains revealed pronounced induction upon mucin exposure (Figure 2B). Similar to *chiA*, deletion of the main regulator ChiR abolished promoter activity of both *chiH* and *chiP*, which encode the holin and the peptidoglycan hydrolase key secretion components of the non-typhoidal T10SS (Figure 2B).

**FIGURE 3:**
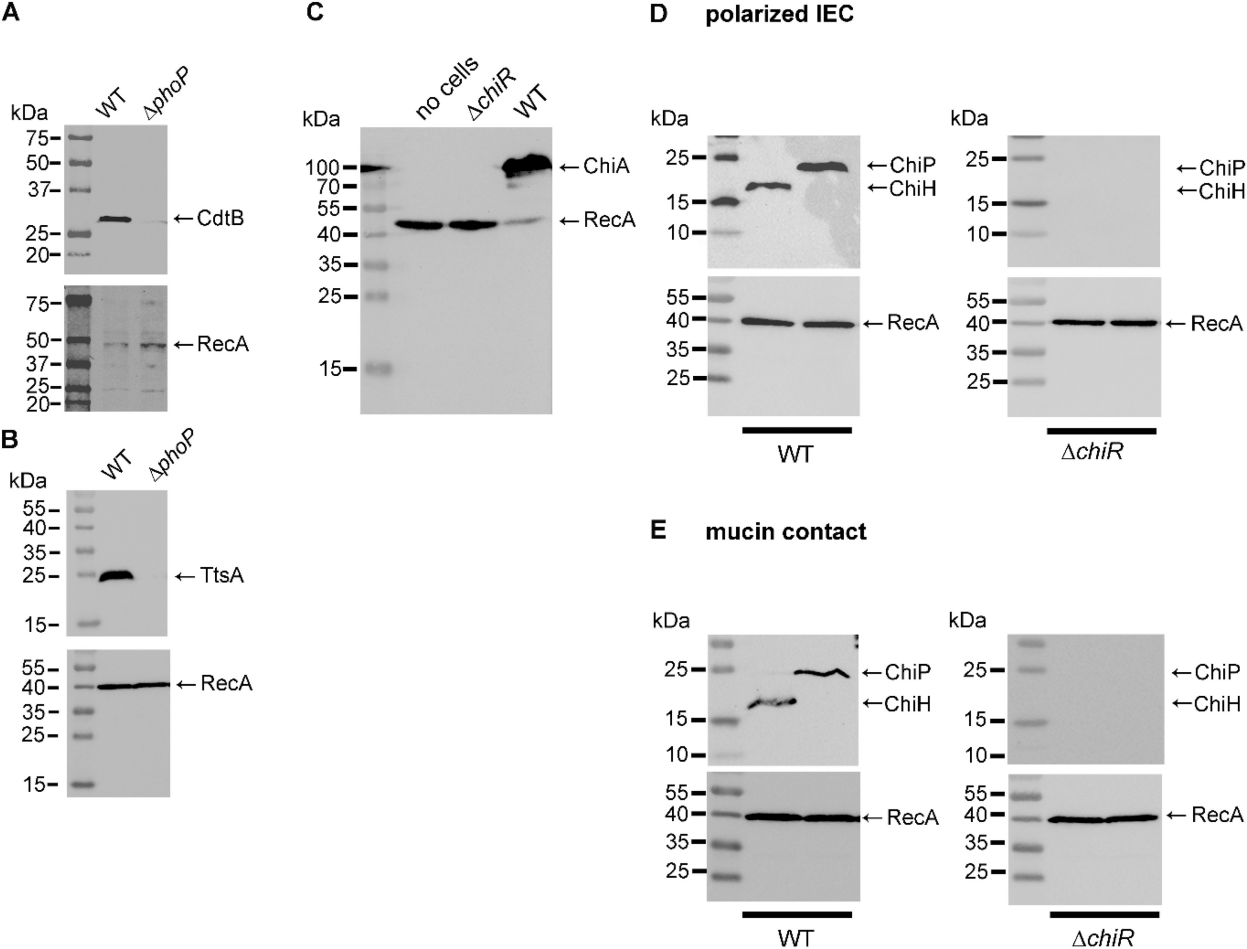
Protein expression analysis of T10SS components and cargo proteins. Detection of **(A)** typhoid toxin (CdtB subunit) and **(B)** TtsA expression by Western blot analysis. Wild-type *S.* Typhi strains carrying chromosomally encoded 3 × FLAG-tagged CdtB or TtsA and the corresponding Δ*phoP* mutant were analyzed. Intracellular bacteria were recovered at 24 h post infection. Western blot analyses were performed using a mouse-α-FLAG antibody (1:10,000). As secondary antibody, rabbit anti-mouse-HRP was used (1:5,000). RecA served as a loading control. **(C–E)** Detection of ChiA, ChiH, and ChiP expression by Western blot analysis. Wild-type *S.* Typhimurium strains carrying chromosomally encoded 3 × FLAG-tagged ChiA, ChiH, or ChiP and the corresponding Δ*chiR* mutants were analyzed. Strains were incubated for 3 h with **(C,D)** polarized CaCo-2 cells or with **(E)** 1% porcine mucin extract (Sigma). Western blot analyses were performed using a mouse-α-FLAG antibody (1:10,000). As secondary antibody, rabbit anti-mouse-HRP was used (1:5,000). RecA served as a loading control.

To verify that induction of T10SS components occurs not only at the promoter level (transcription initiation) but also at the protein level, Western blot analyses were performed to detect TtsA, ChiH, ChiP and the corresponding cargo proteins typhoid toxin (CdtB subunit) and chitinase A (ChiA). For this purpose, strains were generated in which the corresponding proteins were chromosomally tagged at their C-termini with a 3 × FLAG epitope. Upon uptake of *S.* Typhi by HeLa cells, expression of CdtB and TtsA was strongly induced in a PhoP-dependent manner, as no signal was detected in the Δ*phoP* mutant (Figures 3A,B). For the non-typhoidal T10SS, contact with polarized IECs or mucins resulted in ChiR-dependent expression of the chitinase ChiA (Figure 3C), the holin ChiH and the peptidoglycan hydrolase ChiP (Figures 3D,E). Together, these data confirm that, under the tested *in vivo-*like conditions, cell-based infection assays induce both the secreted cargo proteins and the major secretion components at the promoter and protein levels, thereby validating these conditions for subsequent analyses.

### Population-based expression analyses of key components of the T10SS under *in vivo*-like inducing conditions

**FIGURE 4:**
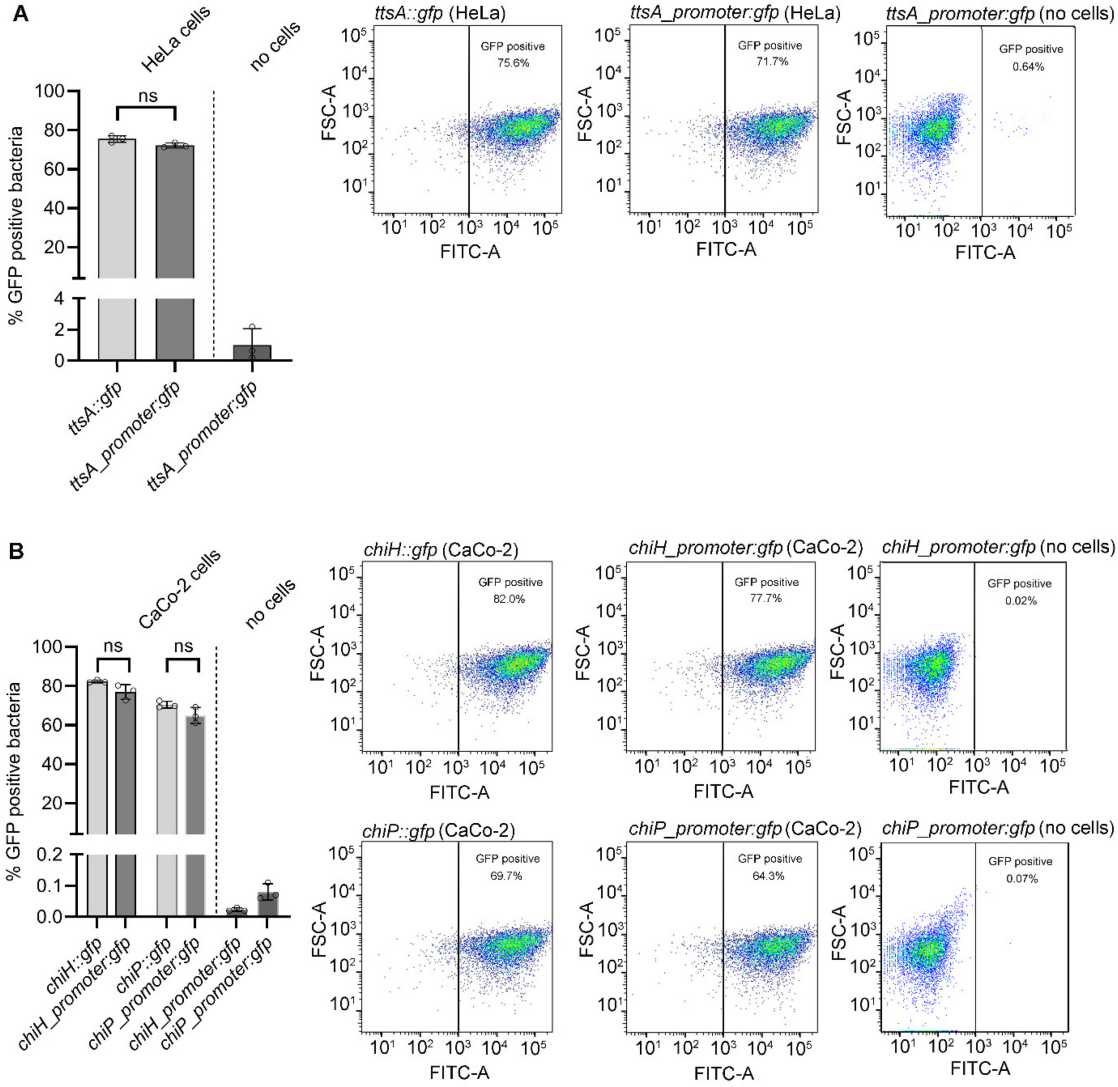
Population level analysis of *Salmonella enterica* expressing T10SS secretion components under *in vivo-*like inducing conditions. **(A)** *S.* Typhi reporter strains carrying either a chromosomal *ttsA:GFP* replacement or a plasmid-based *ttsA* promoter_GFP fusion reporter (*ttsA_promoter:GFP*) were used to infect HeLa cells. Intracellular bacteria were recovered at 24 h post infection, and GFP-positive populations were quantified by flow cytometry. As a control, *S.* Typhi carrying the plasmid-based *ttsA*_promoter*:GFP* construct was incubated in DMEM without HeLa cells (shown in Supplementary Figure 3A). This condition served as a non-inducing control for *ttsA* promoter activity. Data represent three independent experiments (shown in Supplementary Figure 1A) and are shown as mean ± standard deviation. Statistical analysis was performed using an unpaired *t*-test; NS, not significant (*p* > 0.05). **(B)** *S.* Typhimurium reporter strains carrying chromosomal *chiH:GFP* or *chiP:GFP* replacements, or plasmid-based *chiH* or *chiP* promoter_GFP fusion reporters (*chiH_promoter:GFP, chiP_promoter:GFP*), were incubated for 3 h with polarized CaCo-2 cells in DMEM. GFP-positive populations were analyzed by flow cytometry. Again, as a control, *S.* Typhimurium carrying the plasmid-based *chiP*_promoter*:GFP* or *chiH*_promoter*:GFP* constructs were incubated in DMEM without CaCo-2 cells (shown in Supplementary Figure 3A). This condition served as a non-inducing control for *chiP* or *chiH* promoter activities. Data represent three independent experiments (shown in Supplementary Figure 1B) and are shown as mean ± standard deviation. Statistical analysis was performed using an unpaired *t*-test; NS, not significant (*p* > 0.05).

Using chromosomal reporter strains, we performed population-based analyses to determine the proportion of bacteria expressing key T10SS components under the corresponding *in vivo-*like inducing conditions. For the typhoidal T10SS, flow cytometry analyses revealed that about 75% of the intracellular reporter strain (*ttsA:sfGFP*) were fluorescence positive (Figure 4A). Repetition of the experiment using a plasmid-based reporter in which the *ttsA* promoter was fused to *sfGFP* (*ttsA-P:sfGFP*) yielded comparable results, with about 72% of the bacterial population displaying *ttsA*-dependent fluorescence (Figure 4A).

Population analyses of *S.* Typhimurium upon contact with mucin expressing polarized IEC, that induces the non-typhoidal T10SS, showed that reporter strains for *chiH* (*chiH:sfGFP*) and *chiP* (*chiP:sfGFP*) exhibited fluorescence in 82% and 70% of the bacterial population, respectively (Figure 4B). Consistently, plasmid-borne promoter based reporter constructs produced similar results, with 78% of bacteria expressing *chiH* (*chiH-P:sfGFP*) and 64% expressing *chiP* (*chiP-P:sfGFP*), with no significant differences compared to the chromosomal reporters.

Interestingly, flow cytometry control experiments performed in DMEM alone, without host cells, showed that both T10SSs remain completely turned off, indicating very tight regulation by the corresponding regulators PhoP and ChiR, respectively (Figures 4A,B).

Together, these data indicate that, under *in vivo-*like conditions, such as during cell infection or host cell contact, the majority of *Salmonella* cells express key T10SS components, both in *S.* Typhi and *S.* Typhimurium.

### Single-cell dissection of lytic versus non-lytic cargo secretion by the *Salmonella* T10SSs

The T10SS is thought to have originated from lytic bacteriophage cassettes, which are classically used by phages to lyse infected bacterial cells and enable phage release. However, contradictory findings across different bacterial species have left unresolved whether bacteria employing this phage-derived system release cargo proteins passively through cell lysis in minor subpopulations or actively via a non-lytic secretion mechanism.

To address this question in *Salmonella*, reporter strains were constructed in which promoters of secretion components with potential lytic activity were fused to *sfGFP* on a plasmid, while the corresponding genes remained intact in the chromosome, as described before. Using established *in vivo-*like inducing conditions, intracellular uptake for *ttsA* expression and contact with polarized IECs for *chiH* and *chiP* induction, these plasmid-borne reporters exhibited expression patterns and population distributions comparable to those observed with chromosomal reporter constructs (Figure 4). These reporters enabled detection of T10SS induction at the single-cell level. In parallel, fluorescence-positive bacteria were assessed for cell death using the membrane-impermeable dye TO-PRO, which labels cells with compromised cell wall and membrane integrity.

**FIGURE 5:**
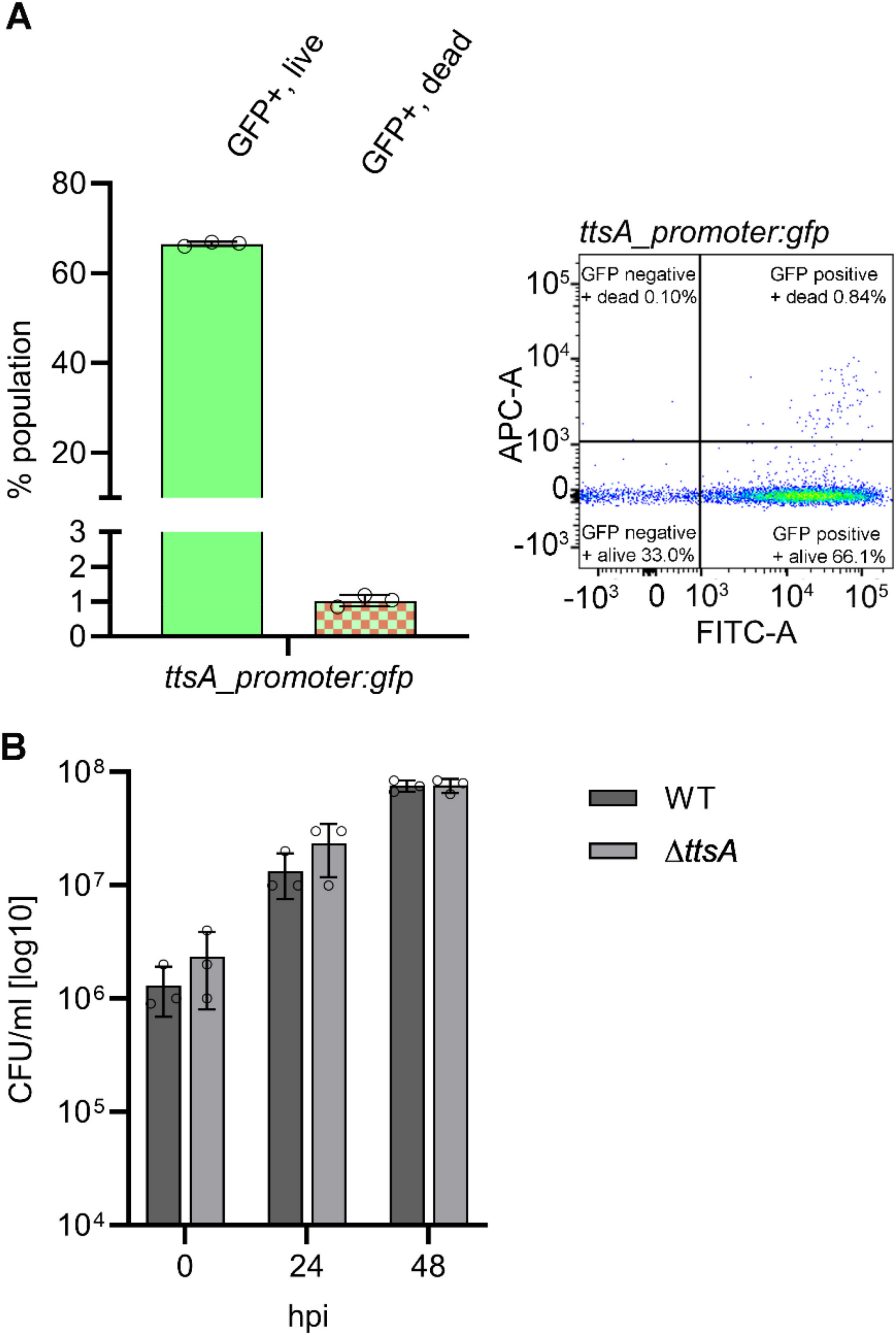
Single cell and population level analysis of *S*. Typhi lysis and survival under T10SS-inducing *in vivo-*like conditions. **(A)** *S.* Typhi wild-type strains carrying a plasmid-based *ttsA*_promoter:GFP reporter were used to infect HeLa cells. Intracellular bacteria were recovered at 24 h post infection (hpi) and stained with the membrane-impermeable nucleic acid dye TO-PRO. GFP-positive/TO-PRO–negative (TtsA-expressing, viable) and GFP-positive/TO-PRO–positive (TtsA-expressing, non-viable) populations were quantified by flow cytometry. Data represent three independent experiments (shown in Supplementary Figure 2A) and are shown as mean ± standard deviation. Corresponding empty vector control experiments are shown in Supplementary Figure 2C. **(B)** Wild-type *S.* Typhi and a clean Δ*ttsA* deletion mutant were used to infect HeLa cells. Bacteria were recovered at 0, 24, and 48 hpi, serially diluted, and plated on LB agar for CFU enumeration. Data represent three independent experiments and are shown as mean ± standard deviation. Statistical analysis was performed using an unpaired *t*-test. No significant differences in bacterial survival were observed between wild-type (secreting) and Δ*ttsA* (non-secreting) strains at any time point (NS, *p* > 0.05).

Of intracellular *S.* Typhi, about 66% of the bacterial population displayed only green fluorescence, indicating active *ttsA* expression in viable cells. Only 0.84% of bacteria exhibited both green and red fluorescence, consistent with cell death during T10SS expression (Figure 5A).

**FIGURE 6:**
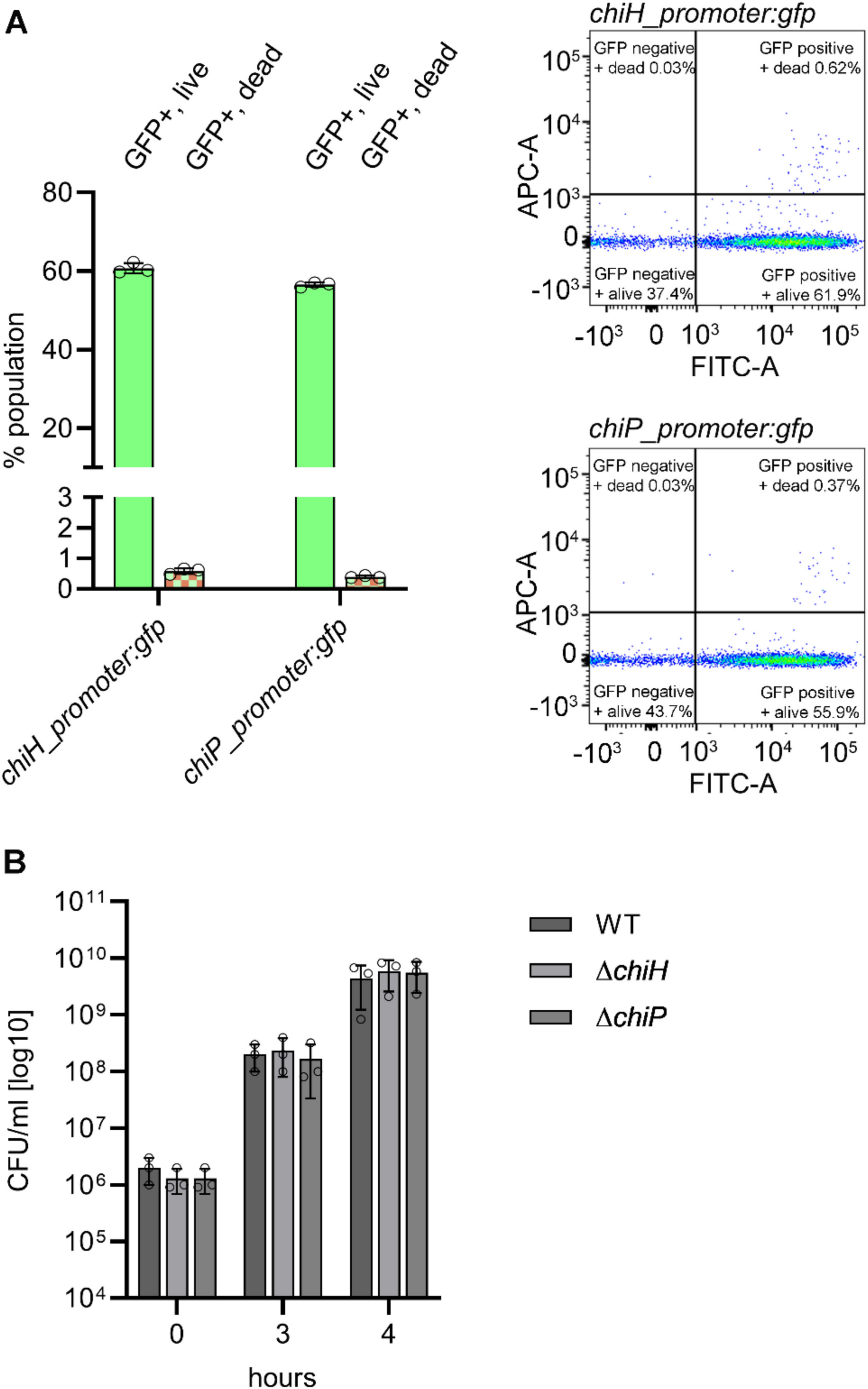
Single cell and population level analysis of *S*. Typhimurium lysis and survival under T10SS-inducing *in vivo-*like conditions. **(A)** *S.* Typhimurium wild-type strains carrying a plasmid-based *chiH*_promoter:GFP or *chiP*_promoter:GFP reporter were incubated with polarized CaCo-2 cells in DMEM. Bacteria were recovered after 3 h and stained with the membrane-impermeable nucleic acid dye TO-PRO. GFP-positive/TO-PRO–negative (ChiH or ChiP-expressing, viable/live) and GFP-positive/TO-PRO–positive (ChiH or ChiP-expressing, non-viable/dead) populations were quantified by flow cytometry. Data represent three independent experiments (shown in Supplementary Figure 2B) and are shown as mean ± standard deviation. Corresponding empty vector control experiments are shown in Supplementary Figure 2D. **(B)** Wild-type *S.* Typhimurium and clean Δ*chiH or*Δ*chiP* deletion mutants were incubated with polarized CaCo-2 cells. Bacteria were recovered at the indicated time points, serially diluted, and plated on LB agar for CFU enumeration. Data represent three independent experiments and are shown as mean ± standard deviation. Statistical analysis was performed using an unpaired *t*-test. No significant differences in bacterial survival were observed between wild-type (secreting) and Δ*chiH or*Δ*chiP* (non-secreting) strains at any time point (NS, *p* > 0.05).

Comparable results were obtained for the non-typhoidal T10SS in *S.* Typhimurium. The majority of bacteria expressing *chiH* (62%) or *chiP* (56%) exhibited green fluorescence exclusively, whereas only a small fraction of the population was positive for red fluorescence (0.62% for *chiH* and 0.36% for *chiP*), indicating minimal cell death (Figure 6A).

These data demonstrate that expression of *ttsA* in *S.* Typhi, as well as *chiH* and *chiP* in *S.* Typhimurium, does not lead to bacterial lysis. Thus, T10SS-mediated secretion in *Salmonella enterica* occurs via a non-lytic mechanism that preserves bacterial viability under *in vivo*–relevant conditions.

Consistently, CFU analyses performed under secretion-inducing conditions such as intracellular localization for *S*. Typhi or in cell contact with polarized Caco-2 cells for *S*. Typhimurium, showed no significant reduction in viability of wild-type bacteria compared with non-secreting Δ*ttsA* (Figure 5B), Δ*chiH*, or Δ*chiP* (Figure 6B) mutants. Together, these results provide strong evidence that the T10SSs function as non-lytic secretion systems in *S. enterica*.

## Discussion

The T10SS, first described in *S.* Typhi and *Serratia marcescens* in 2013 and 2014, respectively, is now recognized as an important mechanism for the secretion of virulence factors across a broad range of bacterial pathogens (Hodak and Galán, 2013; Hamilton et al., 2014). In typhoidal *Salmonella*, T10SS mediates secretion of major toxins, including typhoid toxin and cytolysin A, whereas in non-typhoidal *S. enterica* serovar Typhimurium it is responsible for secretion of the invasion-associated factor chitinase A (Geiger et al., 2018; Krone et al., 2023, 2024).

Comparable functions have been reported in other bacterial species. In *Y. entomophaga*, T10SS mediates secretion of the very large YenTc toxin, while in *S. marcescens* it contributes to chitinase secretion (Hamilton et al., 2014; Sitsel et al., 2024; Feldmüller et al., 2024). T10SS-related mechanisms have also been identified in Gram-positive bacteria such as *Clostridioides difficile*, where a T10SS-like system secretes two Large Clostridial Glycosylating Toxins TcdA and TcdB (Vidor et al., 2022; Govind and Dupuy, 2012; DiBenedetto et al., 2025). Together, these observations indicate that T10SSs are distributed across diverse bacterial species while retaining a conserved functional principle.

At its core, T10SS consists of a minimal protein pair: a holin and a cell wall–degrading enzyme. This enzyme can be an amidase, which cleaves peptidoglycan cross-links, or a muramidase, which hydrolyzes glycan strands; collectively, these enzymes are referred to as peptidoglycan hydrolases (Palmer et al., 2021). Their localized activity transiently disrupts the peptidoglycan barrier, enabling secretion of cargo proteins that would otherwise be unable to traverse the bacterial cell wall. The peptidoglycan hydrolase is itself translocated into the periplasm via the action of the holin, a small inner membrane peptide that forms transient pores in the inner membrane. This core architecture is conserved among all characterized T10SSs, including those of *Yersinia*, *Clostridioides*, *Serratia*, and *Salmonella* (Chowdhury et al., 2025). Some systems additionally encode spanins, which are proposed to contribute to efficient secretion, whereas others, such as the *Salmonella* T10SS, lack spanins yet remain fully functional (Palmer et al., 2021; Chowdhury et al., 2025).

The conserved pairing of a holin with a peptidoglycan hydrolase strongly supports a phage-derived origin for T10SS. In bacteriophages, this protein module mediates host cell lysis to release phage progeny. In *Salmonella*, sequence and domain analyses have traced the evolutionary origin of the T10SS-associated peptidoglycan hydrolase to an N4-Coliphage enzyme (Hodak and Galán, 2013; Stojković and Rothman-Denes, 2007). These hydrolases share a conserved EGGY catalytic domain, underscoring their functional conservation across bacterial and phage systems (Pei and Grishin, 2005).

Despite this shared evolutionary origin, conflicting reports exist regarding whether T10SS functions through a lytic mechanism that passively releases cargo proteins or instead operates as a non-lytic system that actively secretes cargo from viable cells. To address this issue, we investigated T10SS function under physiologically relevant *in vivo-*like inducing conditions in *S. enterica*, focusing on the T10SS of *S.* Typhi and the chitinase A secreting T10SS of *S.* Typhimurium.

In both systems, the central peptidoglycan hydrolase is subject to stringent regulatory control. In *S.* Typhi, expression of *ttsA* depends on PhoP, a key regulator of the PhoP/PhoQ two-component regulatory system that is highly induced within the *Salmonella*-containing vacuole. In *S.* Typhimurium, both the peptidoglycan hydrolase ChiP and the holin ChiH are tightly regulated by ChiR. Unlike the ChiR regulator in *Serratia*, which belongs to the LysR-type transcriptional regulator (LTTR) family, *Salmonella* ChiR is a ToxR-like regulator localized to the inner membrane and containing a defined hydrophobic transmembrane domain (Hamilton et al., 2014; Costa et al., 2019; Krone et al., 2023). This regulator is proposed to sense inducing signals arising from contact with mucin-producing polarized intestinal epithelial cells.

In our study, we demonstrate that, under non-inducing conditions, the key components required for T10SS-mediated secretion that includes the peptidoglycan hydrolases TtsA in *S.* Typhi and the peptidoglycan hydrolase ChiP as well as the holin ChiH in *S.* Typhimurium, are stringently repressed at both the promoter and protein levels. Under these conditions, no expressing bacterial subpopulation is detected. In contrast, upon exposure to inducing conditions, either following uptake into the *Salmonella*-containing vacuole or upon contact with mucin-producing polarized intestinal epithelial cells, these components become clearly detectable at both promoter (transcription initiation) and protein levels.

Population-level analyses further revealed a bimodal expression pattern under *in vivo*–relevant inducing conditions, with approximately 70%–80% of the bacterial population expressing T10SS components, while 20%–30% remained non-expressing. This pattern is consistent with previous observations in *Serratia* (Costa et al., 2019). However, unlike *Serratia*, in which a basal low-expression subpopulation of approximately 1% has been reported (Costa et al., 2019), no such subpopulation was detected in *Salmonella* (Figures 4A,B and Supplementary Figure 3A). Together, these findings indicate that T10SS expression in *Salmonella* is tightly regulated and robustly induced only under specific host-associated conditions.

To determine whether individual T10SS-expressing cells undergo lysis under these physiologically relevant *in vivo-*like conditions, we performed fluorescence-activated flow cytometry analyses at the single-cell level. Cell viability was assessed using the membrane-impermeable nucleic acid stain TO-PRO, which fluoresces red upon entry into dead cells with compromised cell wall and membrane integrity. Across all inducing conditions tested, the TO-PRO–positive population remained well below 1%, which was lower than the level observed under non-inducing conditions, such as incubation in DMEM cell culture medium without host cells, where a death rate of approximately 1.7% was detected (Supplementary Figure 3B). These results indicate that T10SS induction in *S. enterica* occurs without detectable cell lysis. In contrast, previous studies in *Yersinia* and *S. marcescens* strain BS303 relied on artificial overexpression of T10SS components from arabinose-inducible plasmids under low pH conditions (Sitsel et al., 2024). It is likely that such harsh and non-physiological overexpression conditions led to the reported lytic release of cargo proteins in minor bacterial subpopulations. This interpretation is consistent with the evolutionary origin of T10SS from phage lysis cassettes, as deregulated (over)expression of these components outside their native regulatory context is expected to result in membrane and cell wall rupture.

Notably, both T10SSs characterized in *Salmonella* lack spanins, which are present in *Serratia* and *Yersinia* and are required for fusion of the inner and outer membranes during cargo protein release (Sitsel et al., 2024). Spanin-mediated membrane fusion leads to complete cellular disruption and release of intracellular contents. The absence of spanins in *Salmonella* T10SSs may therefore contribute to their non-lytic secretion phenotype. However, this hypothesis requires further investigation, particularly in light of observations in *Serratia* strain DB10, where spanins are present yet no cell lysis was detected under the experimental conditions tested (Hamilton et al., 2014; Palmer et al., 2021; Chowdhury et al., 2025).

Consistent with a non-lytic secretion mechanism, our CFU analyses revealed no difference between secreting and non-secreting bacterial populations under *in vivo-*like inducing conditions in cell-based infection assays. These findings align with previous studies showing that *Salmonella* undergoing T10SS-dependent secretion, either intracellularly or during contact with intestinal epithelial cells, exhibits no reduction in bacterial viability (Geiger et al., 2018; Krone et al., 2023, 2024). Furthermore, the non-expressing (GFP negative) subpopulation, representing approximately 20%–30% of the total population, does not display aberrant cell morphology that would indicate compromised cell wall integrity due to prior secretion events. Such defects would be expected to result in increased TO-PRO staining or altered fluorescence profiles in flow cytometry analyses, neither of which was observed.

In addition to environmental cues, differences in the characteristics of the contributing components may determine whether a T10SS functions lytically or non-lytically. In *S.* Typhi, the peptidoglycan hydrolase TtsA is locally restricted to the bacterial pole in intracellular bacteria (Geiger et al., 2018). Specific C-terminal amino acid residues and protein regions have been shown to be important for this polar localization, as well as for targeting the 3–3 cross-linked peptidoglycan modified by the LD-transpeptidase YcbB, which generates the appropriate substrate for TtsA activity (Geiger et al., 2020). For the chitinase-secreting T10SS in *S.* Typhimurium, analogous mechanisms remain to be explored. Future studies are needed to determine whether similar localization or substrate-targeting features contribute to its non-lytic secretion of chitinase.

Together with our findings we propose that *S. enterica* has evolutionarily repurposed a phage-derived lysis module into a secretion system by imposing stringent regulatory control, eliminating spanin-mediated membrane fusion, and coupling peptidoglycan remodeling to spatial constraints such as polar localization and LD-transpeptidase–dependent cross-link modification. These adaptations ensure that the T10SS functions as an active secretion pathway rather than a terminal lytic mechanism. The cargo proteins highly accumulate in the periplasm, where the peptidoglycan layer has been rendered permeable. Minor disruption of the outer membrane, caused by membrane-disrupting compounds such as antimicrobial peptides or bile salts, or by membrane-active proteins such as those from the saposin or perforin families can efficiently trigger the release of these cargo proteins (Geiger et al., 2018). Additionally, weakening of the peptidoglycan layer may promote the formation of OMVs, which can subsequently mediate cargo protein release, as has been shown for cytolysin A (Krone et al., 2024).

Further elucidation of additional structural and regulatory components will be essential to fully define the molecular mechanisms governing this non-lytic secretion process.

## Data Availability

The original contributions presented in this study are included in the article/Supplementary material, further inquiries can be directed to the corresponding author.
