# Supplementary material for: Non-lytic protein secretion by virulence-associated Type 10 Secretion Systems in Salmonella enterica
Source: Front Microbiol. 2026 Jun 2;17:1809111. doi: 10.3389/fmicb.2026.1809111 (PMC13284979; doi:10.3389/fmicb.2026.1809111)
Supplement: Supplementary file 1 [file Supplementary_file_1.pdf]

Supplementary figures

Supplementary Figure 1

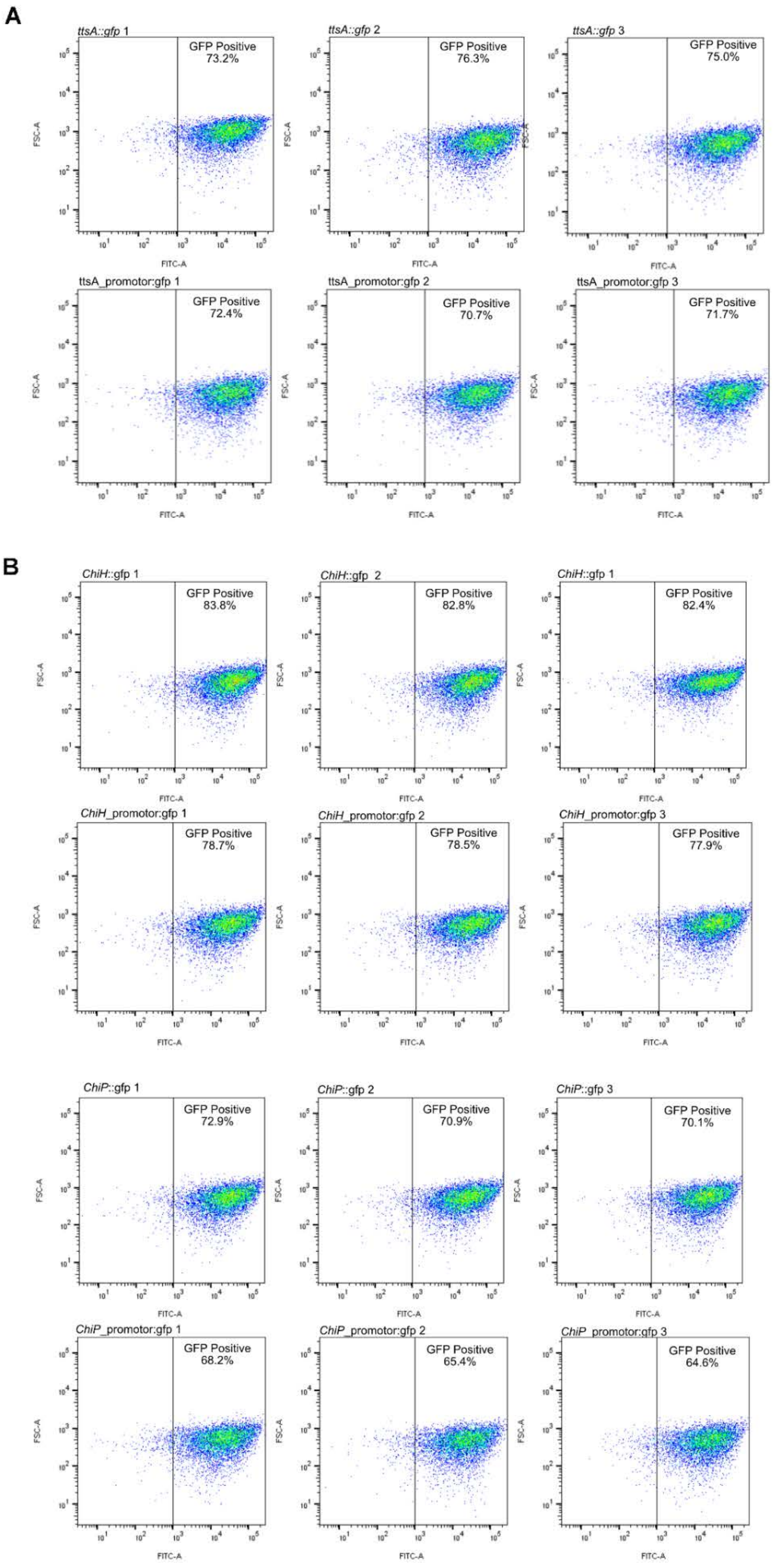

Supplementary Figure 1. Population level analysis of *Salmonella enterica* expressing T10SS secretion components under *in vivo*-like inducing conditions. (A**)** The data shown here represent the three independent experiments that contribute to the quantification shown in Figure 4A. *S.* Typhi reporter strains carrying a chromosomal *ttsA::sfGFP* replacement or a plasmid-based *ttsA* promoter_sfGFP fusion reporter (ttsA_promoter:sfGFP) were used to infect HeLa cells. Intracellular bacteria were recovered at 24 h post infection, and GFP-positive populations were quantified by flow cytometry. (B) The data shown here represent the three independent experiments that contribute to the quantification shown in Figure 4B. *S.* Typhimurium reporter strains carrying chromosomal *chiH::GFP* or *chiP::GFP* replacements, or plasmid-based *chiH* or *chiP* promoter_GFP fusion reporters (*chiH_promoter:GFP, chiP_promoter:GFP*), were incubated for 3 h with polarized CaCo-2 cells in DMEM. GFP-positive populations were analyzed by flow cytometry.

Supplementary Figure 2

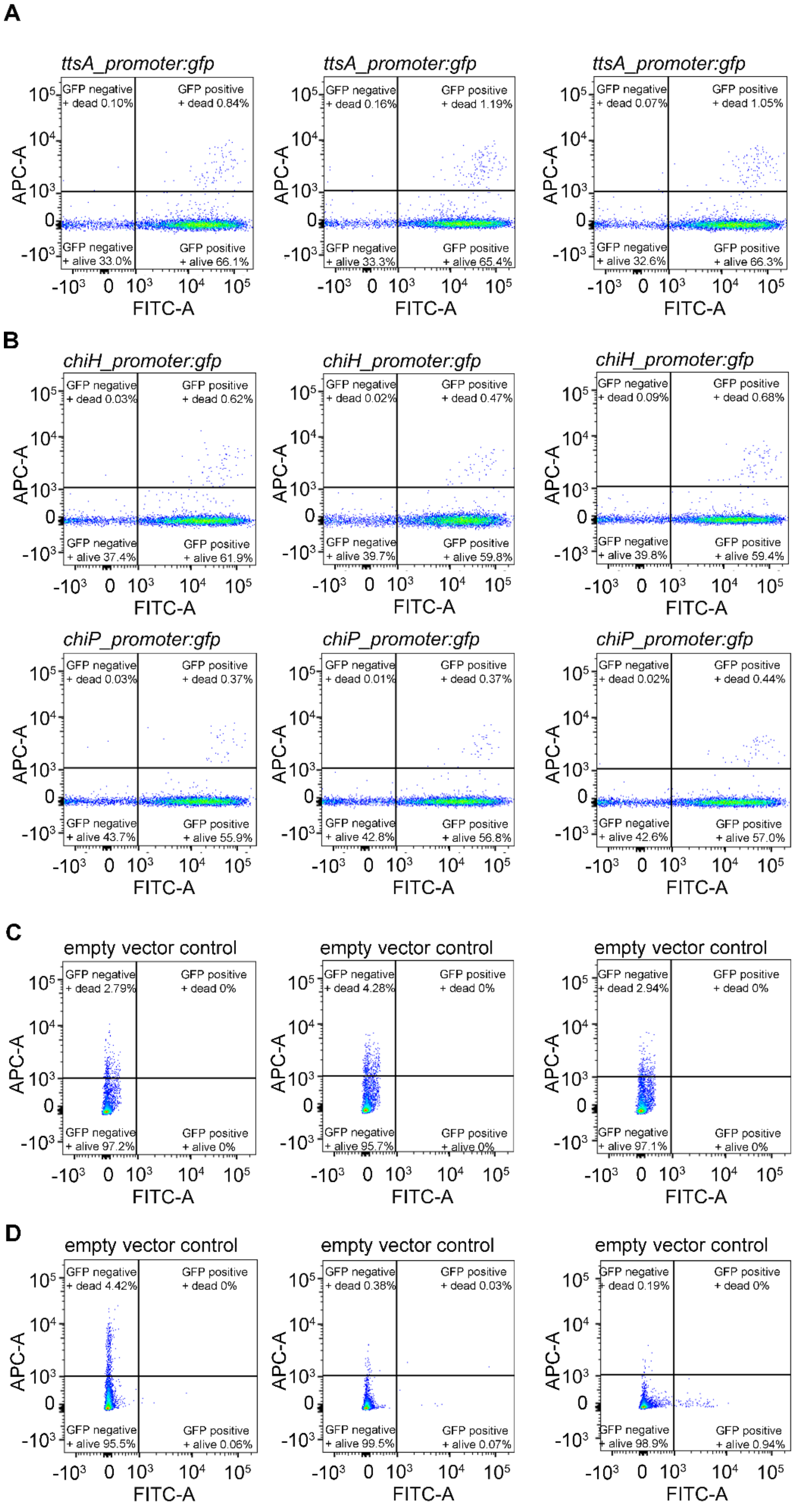

Supplementary Figure 2. Single cell and population level analysis of *S*. Typhi and S. Typhimurium lysis and survival under T10SS-inducing *in vivo*-like conditions. (A-B) The data shown here represent the three independent experiments that each contribute to the quantification shown in Figure 5A and Figure 6A. *S.* Typhi wild-type strains carrying a plasmid-based *ttsA*_promoter:GFP reporter were used to infect HeLa cells. Intracellular bacteria were recovered at 24 h post infection (hpi) and stained with the membrane-impermeable nucleic acid dye TO-PRO. GFP-positive/TO-PRO–negative (TtsA-expressing, viable) and GFP-positive/TO-PRO–positive (TtsA-expressing, nonviable) populations were analyzed by flow cytometry. *S.* Typhimurium wild-type strains carrying a plasmid-based *chiH*_promoter:GFP or *chiP*_promoter:GFP reporter were incubated with polarized CaCo-2 cells in DMEM. Bacteria were recovered after 3 hours and stained with the membrane-impermeable nucleic acid dye TO-PRO. GFP-positive/TO-PRO–negative (ChiH or ChiP-expressing, viable/live) and GFP-positive/TO-PRO–positive (ChiH or ChiP-expressing, nonviable/dead) populations were analyzed by flow cytometry. The data shown here represent the three independent experiments that each contribute to the quantification shown in Figure 5A and Figure 6A. (C-D) Empty vector control experiments were performed using the corresponding empty backbone vectors. In (C), *S.* Typhi harboring the empty vector was used to infect HeLa cells as described in (A), and in (D), *S.* Typhimurium carrying the empty vector was incubated with mucin-producing CaCo-2 cells and processed as described above. The data shown represent three independent experiments.

Supplementary Figure 3

A

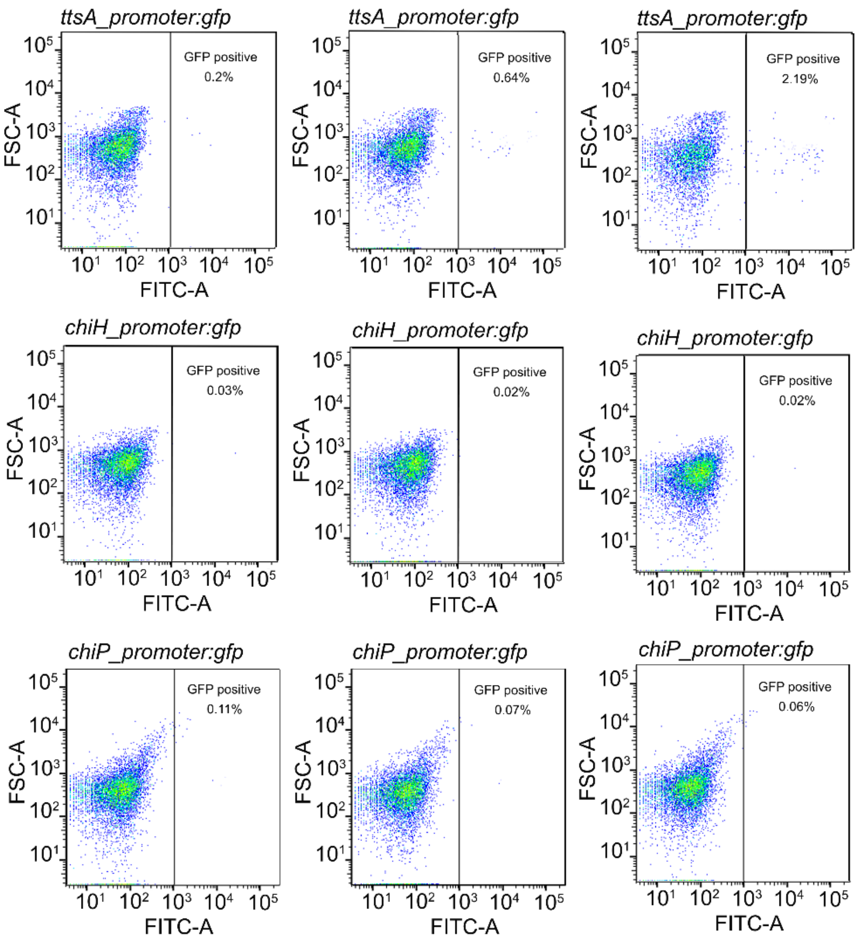

B

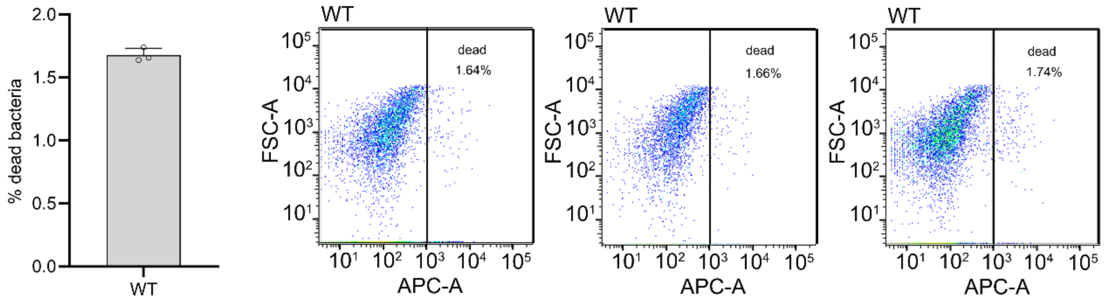

C

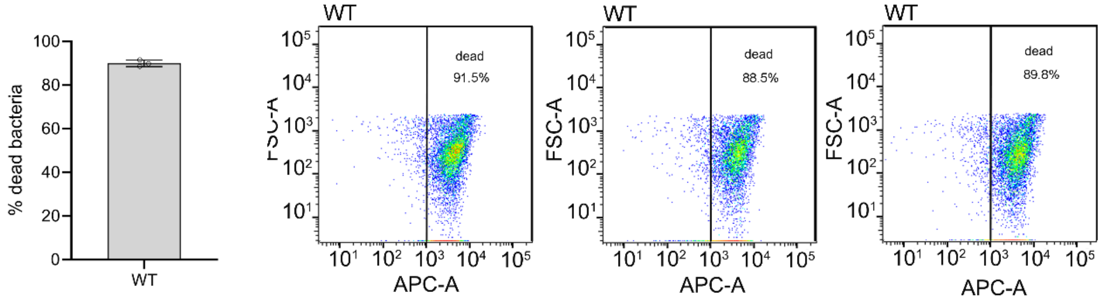

Supplementary Figure 3. (A) Flow cytometry controls for *Salmonella enterica* strains carrying plasmid-based *ttsA* promoter–GFP, *chiH* promoter–GFP, or *chiP* promoter–GFP reporter constructs incubated in DMEM without host cells. These conditions served as non-inducing controls for promoter activity of the indicated genes. The data shown here represent the three independent experiments that each contribute to the quantifications shown in Figure 4A and 4B. (B) Wild-type *Salmonella enterica* serovar Typhimurium grown for 3 h in DMEM without host cells. This condition was used as a control for cell death and analyzed using the membrane-impermeable nucleic acid stain TO-PRO. Cell death was quantified by flow cytometry. Data represent three independent experiments and are shown as mean ± standard deviation. (C) Wild-type *Salmonella enterica* serovar Typhimurium grown for 3 h in DMEM without host cells and subsequently treated with 70% isopropanol at 70 °C for 20 min. This condition served as a positive control for cell death and was analyzed using the membrane-impermeable nucleic acid stain TO-PRO. Cell death was quantified by flow cytometry. Data represent three independent experiments and are shown as mean ± standard deviation.

Supplementary Table 1: Primer used for cloning

chiH-3xF-for	ATCCACTAGTTCTAGAGCGGATGTCTATTCCCAACCATGT
chiH-3xF-rev	ATGACGATGACAAATGATAATTATCATTTGCAGTATCAGG
chiP-3xF-for	ATCCACTAGTTCTAGAGCGGATGAATCCAATTATAGATGG
chiP-3xF-rev	ATGACGATGACAAATGATAAACCAGGCTGAAAAAAGACGC
chiRdel-for	CATTTCTAAAGGAAGACGTTGCGAAGGAAAGGGAAGATGA
chiRdel-rev	TCATCTTCCCTTTCCTTCGCAACGTCTTCCTTTAGAAATG
stm0015del-for	AATGATTTCGGAGTGTTAAATTATCATTTGCAGTATCAGG
stm0015del-rev	CCTGATACTGCAAATGATAATTTAACACTCCGAAATCATT
stm0016del-for	CTAAGCGAAGGAAAGGGAAGACCAGGCTGAAAAAAGACGC
stm0016del-rev	GCGTCTTTTTTCAGCCTGGTCTTCCCTTTCCTTCGCTTAG
stm0015::sfGFPclean-for	TCATTTGTTTGTATCTGCAG
stm0015::sfGFP clean-rev	GTAAAAGGCTCACGGATGAGCCTGATACTGCAAATGATAATTATTTATACAGTTCATCCA
stm0016::sfGFP clean-for	TCTCATACAATATAGTCACCACA
stm0016::sfGFP clean-rev	CTCATCCGTGAGCCTTTTACGCGTCTTTTTTCAGCCTGGTTTATTTATACAGTTCATCCA
ttsA-3xF-clean-for	ATCTTACCCGTAATCAAGCATTAAA
ttsA-3xF-clean-rev	ATGCTATCATCACAAACCGCTATAGTGGTAAGTAAGGGAATTACTATTTATCGTCGTCAT
PhoP-clean-Del-for	TGAAGGGCGTCAGCAAGCTGGAAGT
PhoP-clean-Del-rev	CGCAGCGACAGCGGCAGAAAATGGCGAGCAAATTTATTCACTCTTCTCCCTTGTGTTAAC
ttsA::sfGFP clean-for	GCAAAGAGAGTATGTTTCCA

ttsA::sfGFP clean-rev	ATGCTATCATCACAAACCGCTATAGTGGTAAGTAAGGGAATTATTTATACAGTTCATCCA
ttsa-cleandel-for	AAAGGCCGTGGCGCTAGTTT
ttsa-cleandel-rev	ATGCTATCATCACAAACCGCTATAGTGGTAAGTAAGGGAAAATAACTCCTTCTAATATTA
pTG112-ttsA-P-for	TGGGCGCCCGATAAGCTTAATTACCCGAGAATATTCAAAA
pTG112-ttsA_P-rev	CCTTTGCGCATCAACCTCCTAATGACTCCTTCTAATATTA
pTG113-stm0015-P-for	TGGGCGCCCGATAAGCTTAATATCAGCGAACTTCTGTTTA
pTG113-stm0015-P-rev	CCTTTGCGCATCAACCTCCTTTTAACACTCCGAAATCATT
pTG114-stm0016-P-for	TGGGCGCCCGATAAGCTTAATTTCAAATTTCCTTTTACGT
pTG114-stm0016-P-rev	CCTTTGCGCATCAACCTCCTCTTCCCTTTCCTTCGCTTAG
pTG110-sfGFP-for	CGATTAAGTTGGGTAACGCCATGCGCAAAGGCGAAGAACT
pTG110-sfGFP-rev	GTCGTGACTGGGAAAACCCTTTATTTATACAGTTCATCCA
